# Post-Traumatic Diabetes: Focus on Psychiatric Trauma

**DOI:** 10.1192/j.eurpsy.2025.1194

**Published:** 2025-08-26

**Authors:** C. Bey, T. Ach, A. Ben Abdelkarim

**Affiliations:** 1 University of Sousse, Faculty of Medicine of Sousse, 4000; 2 Endocrinology department, Farhat Hached University Hospital of Sousse, Sousse, Tunisia

## Abstract

**Introduction:**

Physical and/or psychological trauma may contribute to the onset of type 1 diabetes. Forensic medicine experts recognize post-traumatic diabetes in rare cases that meet specific criteria, including the severity of the trauma, its occurrence within a short timeframe before diabetes onset, and the absence of prior diabetes indicators.

**Objectives:**

This study aims to describe the clinical and biological characteristics of post-traumatic diabetes, with particular emphasis on the psychiatric aspects.

**Methods:**

This retrospective, cross-sectional study analyzed cases of patients hospitalized for acute diabetes following physical or psychological trauma. The diagnosis of post-traumatic diabetes was based on the criteria of French forensic experts: acute autoimmune diabetes, trauma occurring within six months before diagnosis, and no signs suggesting pre-existing diabetes. Autoimmune involvement was confirmed by the presence of anti-pancreatic antibodies, specifically anti-glutamic acid decarboxylase (GAD) and/or anti-tyrosine phosphatase IA2.

**Results:**

The study included 10 patients (8 men and 2 women) aged between 17 and 47 years (mean age 29.5±11 years). Family history of autoimmune disease was present in 40% of cases, and type 2 diabetes in 60%. The average body mass index was 24±6 kg/m², with obesity in 30% of cases. The mean blood glucose at admission was 14.54±3.48 mmol/L, and the average HbA1C was 6.51±0.56%. Anti-GAD antibodies were present in all patients, while anti-IA2 antibodies were detected in 20%. Clinically, 60% of patients presented with ketosis, and 40% with ketoacidosis. Psychiatric trauma, including grief in 3 patients and divorce in 2 patients, was the triggering factor in 50% of cases. All patients required insulin therapy upon admission, with obese patients receiving additional metformin.

**Conclusions:**

This study supports the hypothesis of post-traumatic diabetes, particularly in cases of severe psychiatric trauma. Although scientific literature remains inconclusive, the role of stress in the onset of diabetes warrants further investigation due to the heterogeneity of findings regarding stress episodes preceding diabetes development.

**Disclosure of Interest:**

None Declared

